# Molecular Mechanism of Artemisinin Against *Aeromonas hydrophila* LP-2 and the Combined Effect of Gallic Acid

**DOI:** 10.3390/biology15212008

**Published:** 2026-11-05

**Authors:** Ruixiang Shi, Xin Chen, Yuheng Liang, Yixin Huang, Yixin Xie, Huiyan Xie, Bridgitta Bianca Goh, Xiangmin Lin, Lingli Lian

**Affiliations:** 1 School of Life Sciences, Fujian Agriculture and Forestry University, Fuzhou 350002, China; 2 Fujian-Taiwan Science and Technology Cooperation Base of Fujian Province, Innovation Base for Germplasm Innovation and Green Cultivation of Characteristic Crops, Fuzhou 350002, China; 3 Collaborative Innovation Center for Germplasm Creation and Green Cultivation of Fujian-Taiwan Characteristic Crops, Fuzhou 350002, China; 4 Key Laboratory of Marine Biotechnology of Fujian Province, Institute of Oceanology, Fujian Agriculture and Forestry University, Fuzhou 350002, China

**Keywords:** artemisinin, *Aeromonas hydrophila*, antibacterial mechanism, gallic acid, proteome

## Abstract

*Aeromonas hydrophila* poses a growing threat in aquaculture due to high mortality and increasing antibiotic resistance, highlighting an urgent need for novel antimicrobial agents. Artemisinin (ART), a natural bioactive compound, exhibits antibacterial activity against *A. hydrophila*, yet its underlying mechanism remains poorly understood. In this study, quantitative proteomics was employed to elucidate the antibacterial mechanism of ART against *A. hydrophila* LP-2 and to evaluate its combined inhibitory effect with gallic acid (GA). The results showed that ART inhibited *A. hydrophila* in a concentration-dependent manner. At concentrations of 400 μg/mL and above, ART reduced biofilm mass independently of planktonic growth and also induced cell membrane damage and intracellular reactive ROS accumulation. Proteomic analysis indicated that ART significantly perturbed multiple metabolic pathways, including amino acid metabolism, redox metabolism, ABC transporters, and quorum sensing. Furthermore, the combination of ART and GA exhibited a pronounced antibacterial effect, which was closely associated with massive accumulation of intracellular ROS. Mechanistically, oxidative stress-related genes (*orf00650*, *orf00652*) were significantly upregulated, whereas the efflux transporter gene (*orf00659*) was markedly downregulated, suggesting that the combined effect is jointly driven by enhanced oxidative damage and impaired drug efflux. Hemocompatibility test showed that ART caused no detectable hemolysis. This study reveals the multifaceted antibacterial mechanism of artemisinin against *A. hydrophila* LP-2 and demonstrates that gallic acid significantly enhances its antibacterial activity in this strain, providing a theoretical basis for the further development of artemisinin as a potential antibacterial agent against *A. hydrophila*.

## Simple Summary

Aquaculture is a rapidly growing food industry, but it is threatened by a widespread bacterium called *Aeromonas hydrophila*, which causes severe disease and death in farmed fish. Farmers often rely on antibiotics to control this bacterium, but overuse has made many of these drugs ineffective and has left harmful residues in the environment. There is therefore an urgent need for safer alternatives. We tested if artemisinin, a natural plant compound already known as an antimalarial drug, can inhibit this harmful bacterium and elucidated its mechanism. We found that artemisinin blocked the bacterium’s ability to form protective slimy layers called biofilms, damaged the bacterial cell surface and triggered harmful reactive oxygen species inside the cells. We also found that combining artemisinin with gallic acid, another natural compound, greatly boosted the killing effect by increasing this internal oxidative stress and by blocking the bacterium’s ability to pump the drug out. Importantly, artemisinin did not damage red blood cells, suggesting a low hemolytic risk. Our findings support the development of artemisinin-based natural treatments to control bacterial disease in fish farming, helping to reduce reliance on antibiotics and protect both the environment and public health.

## 1. Introduction

*Aeromonas hydrophila* is a Gram-negative pathogenic bacterium widely distributed in aquatic ecosystems. It causes motile aeromonad septicemia (MAS) in fish, leading to high mortality and substantial economic losses in aquaculture, and poses a zoonotic risk to humans, causing acute gastroenteritis, wound infections, and sepsis [1,2]. In recent years, intensive and high-density aquaculture practices have significantly increased the risk of *Aeromonas* colonization and disease outbreaks. Consequently, the global prevalence of MAS in freshwater aquaculture has been rising annually [3,4]. Current prevention and control measures rely predominantly on antibiotics. However, the long-term irrational use of antibiotics has not only driven the proliferation of multidrug-resistant strains but also led to serious drug residues and ecological risks. Therefore, there is an urgent need to identify and develop safe, effective, and novel antimicrobial agents.

Compared with conventional antibiotics, natural products possess advantages such as multi-target interference, low propensity for inducing drug resistance, and strong environmental compatibility, making them an increasingly important source of novel agents to control *A. hydrophila*. Current research has focused primarily on plant-derived bioactive compounds, including phenolic acids (e.g., tannic acid), flavonoids (e.g., genistein), and phenylpropanoids (e.g., cinnamaldehyde and resveratrol). These compounds can inhibit *Aeromonas* infection by attenuating bacterial virulence phenotypes rather than through direct bactericidal mechanisms [5]. Furthermore, studies have shown that Chinese herbal medicines containing flavonoids, terpenoids, and other active ingredients can enhance the immune function of cultured fish, thereby indirectly enhancing their resistance to motile aeromonad septicemia, demonstrating their potential as environmentally friendly and low-cost alternative strategies for disease control [6]. Among these natural products, terpenoids stand out due to their remarkable structural diversity and broad biological activities, and have increasingly become a new research focus [7,8].

Among terpenoids, artemisinin (ART), which is characterized by a unique endoperoxide bridge structure, has been preliminarily demonstrated to inhibit the quorum sensing system and virulence factors of *A. hydrophila* in vitro [9]. Furthermore, in vivo experiments have confirmed that ART ethanol extract can enhance the resistance of Nile tilapia (*Oreochromis niloticus*) to *A. hydrophila* infection by boosting their innate immune system [10]. However, other studies have shown that ART aqueous extract exhibits relatively weak antibacterial activity against *Aeromonas veronii*, suggesting that the efficacy of ART may vary depending on the extraction method or the specific bacterial strain [11]. This discrepancy may also arise from the low ART content in aqueous extracts. Therefore, it is essential to clarify the inhibitory effects and action characteristics of ART against different *Aeromonas* strains, so as to provide a more robust evidence base for its development and application.

With respect to the elucidation of the molecular mechanisms underlying the antibacterial effects of ART, Zhai et al. conducted a comprehensive review and found that the antibacterial mechanisms of ART and its derivatives exhibit typical multi-target characteristics, involving six major aspects: inhibition of efflux pump activity, disruption of cell membrane integrity, inhibition of biofilm formation, suppression of resistance gene expression, induction of reactive oxygen species (ROS) accumulation, and interference with metabolic activities [12,13]. Notably, ART combined with antibiotics, encapsulated in chitosan nanoparticles, or co-treated with copper ions (Cu^+^) has demonstrated significant synergistic antibacterial effects, the molecular basis of which primarily involves transcriptional inhibition of efflux pump- and biofilm-related genes, as well as a marked increase in intracellular ROS levels [14,15,16]. Nevertheless, these mechanistic studies have largely focused on pathogenic bacteria such as *Escherichia coli*, *Pseudomonas aeruginosa*, *Staphylococcus aureus*, *Vibrio cholerae*, and *Mycobacterium tuberculosis*, while research on the mechanisms of ART against *A. hydrophila* remains limited. Existing reports have confirmed that artemisinin can target and inhibit the AhyI synthase of *A. hydrophila*, and at sub-inhibitory concentrations, it can impair biofilm formation and motility; however, the specific molecular mechanisms have not been systematically elucidated [9].

To further investigate the molecular mechanisms by which ART inhibits *A. hydrophila*, this study evaluated its in vitro antibacterial activity, and subsequently examined its effects on key phenotypes including biofilm formation ability, cell membrane permeability, and reactive oxygen species (ROS) levels. Furthermore, quantitative proteomics was employed to elucidate the underlying molecular mechanisms, and to identify key metabolic modules and differentially expressed proteins. In addition, the antibacterial effects of ART in combination with relevant inhibitors were investigated. The findings of this study will provide a theoretical basis for clarifying the inhibitory effects and intrinsic mechanisms of ART against *A. hydrophila*, and for advancing its potential application in the prevention and control of *Aeromonas* infections, pending further in vivo evaluation in aquaculture settings.

## 2. Materials and Methods

### 2.1. Bacterial Strains and Culture Conditions

Nine aquatic pathogenic bacterial strains were used to determine the antibacterial activity of artemisinin: *A. hydrophila* LP-2, *A. hydrophila* ATCC 7966, *Vibrio proteolyticus*, *V. parahaemolyticus*, *V. alginolyticus*, *V. harveyi*, *Photobacterium damselae*, *Pseudomonas fluorescens*, and *P. plecoglossicida*. Among them, *A. hydrophila* LP-2 was selected for subsequent mechanistic analysis. All strains were cultured in freshly prepared Luria–Bertani (LB) broth with shaking at 200 rpm at 30 °C [17].

### 2.2. Growth Curve Determination

Bacterial growth curves were measured using an automated growth curve analyzer [18]. Overnight cultures of the test strains were inoculated into fresh LB medium at a ratio of 1% (*v*/*v*), and ART (artemisinin, dissolved in anhydrous ethanol at 20 mg/mL stock) was added to final concentrations of 0 (control), 200, 400, and 800 μg/mL. Control groups received an equal volume of ethanol, and a solvent control confirmed that ethanol alone did not affect bacterial growth at concentrations matching those used for 200 and 400 μg/mL ART, whereas a minor effect was observed at the concentration corresponding to 800 μg/mL. After thorough mixing, 300 μL aliquots were transferred into a sterile 96-well microplate. The plate was sealed and placed in the analyzer, which recorded the absorbance at 600 nm every hour for 16 h at 30 °C. Three biological replicates were performed for each group.

### 2.3. Physiological Phenotype Assays

#### 2.3.1. Biofilm Formation Assay

Biofilm formation was assessed using the crystal violet staining method [19]. Overnight cultures of strain LP-2 were inoculated into fresh LB broth at a ratio of 1% (*v*/*v*), and ART stock solution was added to final concentrations of 0 (control), 200, 400, and 800 μg/mL. Aliquots of 200 μL of the bacterial suspension were transferred to each well of a 96-well plate and incubated statically at 30 °C for 16 h. The culture medium was then removed, and the wells were washed three times with sterile distilled water. After fixation with 200 μL of methanol for 15 min, the wells were washed again. The adherent cells were stained with 200 μL of 0.1% crystal violet for 20 min at room temperature, washed, and destained with 220 μL of 95% ethanol for 10 min. Finally, the absorbance (OD_595_) was measured using a microplate reader. Three biological replicates were performed for each group.

#### 2.3.2. Membrane Permeability Assay

Cell membrane permeability was assessed using propidium iodide (PI) staining [20]. Overnight cultures of strain LP-2 were inoculated into fresh LB broth and grown with shaking at 200 rpm at 30 °C until the OD_600_ reached 0.6. Cells were harvested by centrifugation at 8000 rpm for 5 min, washed twice with PBS, and resuspended in PBS. PI was added to a final concentration of 10 μM and the cells were incubated at 30 °C for 30 min in the dark. ART stock solution was then added to final concentrations of 0 (control), 200, 400, and 800 μg/mL. Aliquots of 200 μL of the mixture were transferred to a black 96-well plate and incubated at 30 °C for 1 h in the dark. Finally, the fluorescence intensity was measured using a multifunctional microplate reader with excitation and emission wavelengths of 535 nm and 615 nm, respectively. Three biological replicates were performed for each group.

#### 2.3.3. Intracellular Reactive Oxygen Species (ROS) Assay

Intracellular ROS levels were detected using the 2′,7′-dichlorofluorescein diacetate (DCFH_2_-DA) fluorescent probe [21]. Overnight cultures of strain LP-2 were inoculated into fresh LB broth at a ratio of 1% (*v*/*v*), and ART stock solution was added to final concentrations of 0 (control), 200, 400, and 800 μg/mL. The cultures were incubated with shaking at 200 rpm at 30 °C for 3 h. Cells were then harvested by centrifugation at 8000 rpm for 5 min, washed three times with PBS, and resuspended in PBS. These washing steps were designed to remove extracellular free ART, so that the bacteria were resuspended in a drug-free PBS system before probe addition. DCFH_2_-DA working solution was added to a final concentration of 10 μM. After thorough mixing, the samples were incubated in the dark for 30 min. The cells were centrifuged at 8000 rpm for 5 min and washed again with PBS to remove the unbound probe. Thus, from probe addition to fluorescence measurement, the bacteria were maintained in a drug-free PBS system, ensuring that the measured fluorescence reflected intracellular oxidation rather than extracellular probe oxidation by ART. Aliquots of 200 μL of the cell suspension were transferred to a black 96-well plate, and the fluorescence signal was measured using a microplate reader with excitation and emission wavelengths of 488 nm and 525 nm, respectively. Three biological replicates were performed for each group.

### 2.4. Quantitative Proteomics Analysis

#### 2.4.1. Protein Sample Preparation

Overnight cultures of strain LP-2 were inoculated into fresh LB broth at an inoculum of 1% (*v*/*v*). ART stock solution was added to the treatment group at a final concentration of 200 μg/mL, while the control group received no drug. Both groups were cultured under the same conditions with shaking at 200 rpm at 30 °C until the OD_600_ reached 1.0. Cells were collected by centrifugation at 7000× *g* for 15 min at 4 °C, washed three times with PBS, and resuspended in lysis buffer containing 6 M urea, 2 M thiourea, and 0.1 M Tris-HCl (pH 8.5), supplemented with protease inhibitors. After sonication (30% power, 9 s on, 6 s off, for a total of 15 min), the supernatant was collected by centrifugation at 18,000× *g* for 15 min at 4 °C, and the protein concentration was determined using the Bradford method [22]. Three biological replicates were performed for each group.

#### 2.4.2. LC-MS/MS Analysis

Protein samples were digested with trypsin using in-solution digestion. Briefly, proteins were reduced with 5 mM DTT at 56 °C for 30 min and alkylated with 10 mM IAA for 30 min. Trypsin was added at a protein:enzyme ratio of 100:1, and digestion was performed at 37 °C overnight. The reaction was terminated by adding 1% TFA, and the resulting peptides were desalted using C18 solid-phase extraction columns, dried, and redissolved in mobile phase A (0.1% formic acid in water) with iRT standard peptides. Chromatographic separation was performed on a nanoElute 2 system (Bruker, Bremen, Germany) equipped with an AUR3-15075C18 analytical column (25 cm × 75 μm i.d., 1.7 μm particle size, 120 Å pore size, IonOpticks, Melbourne, Australia). Peptides were eluted with a 60-min gradient at a flow rate of 300 nL/min, and the column temperature was maintained at 55 °C. The nano-LC was coupled online to a TimsTOF Pro2 mass spectrometer (Bruker Daltonics, Bremen, Germany) equipped with a nano-electrospray ion source. Data acquisition was performed in diaPASEF mode. The scan range was set to 349–1229 *m*/*z*, and the isolation window width was 40 Da. In PASEF MS/MS scans, collision energy increased linearly from 20 eV (1/K_0_ = 0.6 Vs/cm^2^) to 59 eV (1/K_0_ = 1.6 Vs/cm^2^). Data were searched using Spectronaut 18.0 (Biognosys AG, Schlieren, Switzerland) with default parameters. The search was performed against the complete proteome database of *A. hydrophila* LP-2. Precursor and protein FDR thresholds were both set to 1.0%, and the decoy database was generated using a mutated strategy. For DIA data quantification, Spectronaut extracted fragment ion chromatograms using local cross-correlation, and protein intensities were calculated as the average peak area of the top 3 peptides per protein group with <1.0% FDR. Protein intensities were normalized by median normalization across runs [23].

#### 2.4.3. Mass Spectrometry Data Analysis

Sample reproducibility and differential significance were assessed for the proteomic quantification results. Sample reproducibility was first evaluated by Pearson correlation analysis, combined with principal component analysis (PCA) to examine the clustering of the control and treatment groups. Differentially expressed proteins (DEPs) were then screened using the criteria of a fold change >2 or <0.5 with a *p*-value < 0.05. GO functional and KEGG pathway enrichment analyses were performed using the DAVID online platform (https://david.ncifcrf.gov/, accessed on 30 October 2024). The protein–protein interaction (PPI) network was generated via the STRING database (version 12.5, https://string-db.org, accessed on 30 October 2024) using default settings that combine experimentally determined interactions and computationally predicted associations. Network visualization and hub protein identification were performed in Cytoscape 3.10.2.

### 2.5. RT-qPCR Validation

*A. hydrophila* cells treated with 200 μg/mL ART and untreated control cells were collected, and total RNA was extracted using the E.Z.N.A.^®^ Bacterial RNA Kit (R6950-01, Omega Bio-tek, Norcross, GA, USA). Total RNA was reverse-transcribed into cDNA using the First-strand cDNA Synthesis Mix for qPCR (F0202, LabLead, Guangzhou, China). Gene-specific qPCR primers were designed based on the sequences of the differentially expressed genes identified by proteomics (primer sequences are listed in Table S1), and DNA gyrase subunit B was used as the reference gene. qPCR amplification was performed with 2× Realab Green PCR Fast Mixture (R0202, LabLead, Guangzhou, China) using the SYBR-Green dye method on a LightCycler^®^ 96 Instrument (Roche, Basel, Switzerland), followed by melting curve analysis to verify primer specificity. The amplification program was as follows: an initial denaturation step at 95 °C for 30 s, followed by 40 cycles of 95 °C for 10 s, 60 °C for 30 s, with a post-amplification melting curve ranging from 65 °C to 95 °C. Three biological replicates were performed for each treatment. The relative mRNA expression of each gene was calculated using the 2^−ΔΔ^^Ct^ method.

### 2.6. Antibacterial Effects of Artemisinin in Combination with Other Compounds

The antibacterial effects of ART in combination with TPEN (N,N,N′,N′-tetrakis (2-pyridylmethyl) ethylenediamine), BIP (2,2′-bipyridine), and GA (gallic acid) were analyzed by growth curve assays. Overnight cultures of strain LP-2 were inoculated into fresh LB broth at an inoculum of 1% (*v*/*v*). A blank control group, single-drug groups (ART, TPEN, BIP, and GA alone), and combination groups were established. The cultures were incubated with shaking at 200 rpm at 30 °C, and the OD_600_ was monitored at regular intervals to construct growth curves. The concentrations of TPEN and BIP were 50 μg/mL and 31.2 μg/mL, respectively, while GA was tested at 100 and 200 μg/mL. ART was used at 200 μg/mL in all combination groups. In addition, the intracellular ROS accumulation induced by the combined treatment of GA and ART was determined using the DCFH_2_-DA fluorescent probe, and the fluorescence signal was read with a microplate reader (excitation/emission: 488/525 nm) to assess the level of oxidative stress. Three biological replicates were performed for each group.

### 2.7. In Vitro Hemolytic Toxicity Evaluation of Artemisinin

The hemolytic activity of ART was evaluated by a red blood cell hemolysis assay [24]. Fresh sheep blood, a standard model for in vitro hemolysis testing, was washed three times with ice-cold sterile PBS and prepared as an 8% erythrocyte suspension in PBS. ART was added to final concentrations of 0, 50, 100, 200, 400, and 800 μg/mL, with PBS buffer and 0.2% Triton X-100 solution serving as the negative and positive controls, respectively. The mixtures were incubated at 37 °C in the dark for 1 h and then centrifuged at 1000× *g* for 5 min. The supernatant was collected, and the absorbance was measured at 576 nm using a microplate reader. The hemolysis rate was calculated using the following formula: Hemolysis rate (%) = (ODsample − ODnegative)/(ODpositive − ODnegative) × 100%. Three biological replicates were performed for each group.

## 3. Results

### 3.1. Antibacterial Activity of Artemisinin

**Figure 1:**
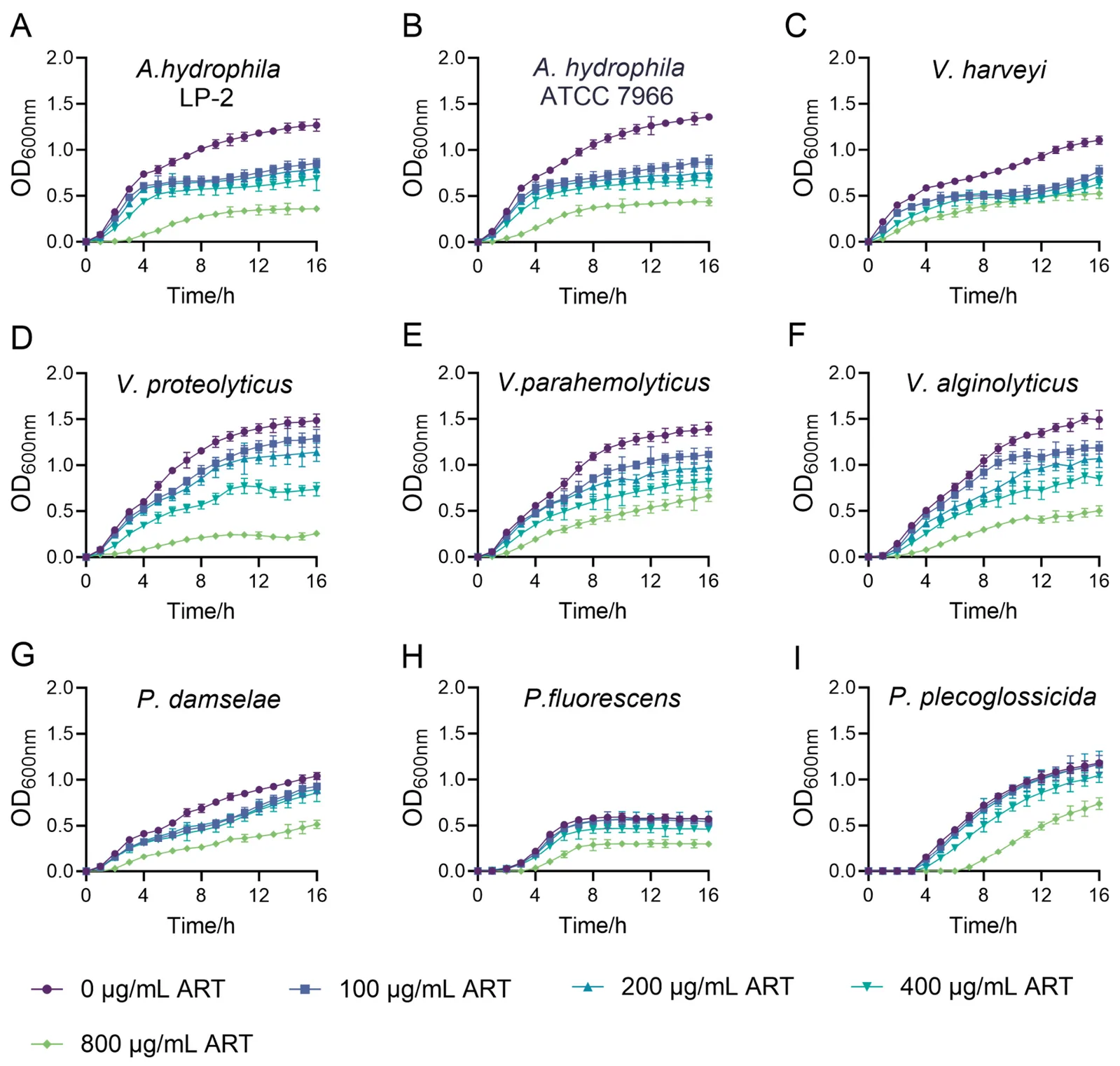
Inhibitory effects of artemisinin on the growth of nine pathogenic bacteria. (**A**) *A. hydrophila* LP-2; (**B**) *A. hydrophila* ATCC 7966; (**C**) *V. harveyi*; (**D**) *V. proteolyticus*; (**E**) *V. parahemolyticus*; (**F**) *V. alginolyticus*; (**G**) *P. damselae*; (**H**) *P. fluorescens*; (**I**) *P. plecoglossicida*. OD_600nm_ was detected at indicated time points. Data represent the mean ± standard deviation (SD) of three independent biological replicates.

To determine the inhibitory activity of artemisinin (ART) against aquatic pathogenic bacteria, the growth of nine strains was measured at gradient concentrations of ART. The results showed that, compared with the untreated control, ART exhibited growth inhibitory effects on all tested strains, though the response characteristics varied markedly among different species. ART exhibited the strongest inhibitory activity against *A. hydrophila* LP-2 and ATCC 7966. At 100 and 200 μg/mL, it significantly retarded bacterial growth and reduced the maximum biomass, and this inhibitory effect was further enhanced at 800 μg/mL (Figure 1A,B). In comparison, ART also inhibited *Vibrio proteolyticus*, *V. parahaemolyticus*, *V. alginolyticus*, and *V. harveyi* at low concentrations, but with lower efficacy than against *A. hydrophila* (Figure 1C–F). Among these, inhibition of the former three was typically concentration-dependent, whereas that of *V. harveyi* showed no significant concentration-related enhancement. In contrast, ART exhibited substantially weaker antibacterial activity against *Photobacterium damselae*, *Pseudomonas fluorescens*, and *P. plecoglossicida* (Figure 1G–I). At low and medium concentrations (100, 200, and 400 μg/mL), it only weakly inhibited the growth of *P. damselae* and had almost no effect on the two *Pseudomonas* strains; significant inhibition of all three pathogens was only achieved at a high concentration (800 μg/mL). Since *A. hydrophila* LP-2 was among the most sensitive strains tested, it was chosen as the representative strain to further investigate the antibacterial mechanism of artemisinin.

### 3.2. Inhibitory Effects of Artemisinin on Physiological Phenotypes of A. hydrophila

**Figure 2:**
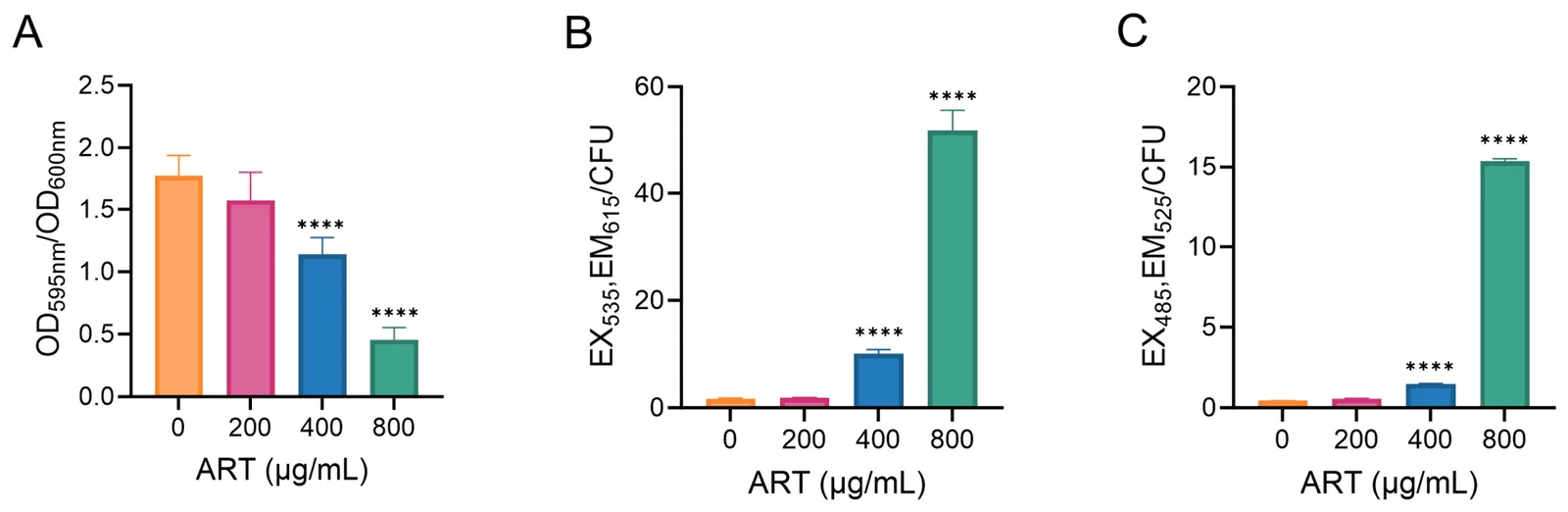
Effects of artemisinin on the physiological phenotypes of *A. hydrophila* LP-2. (**A**) Biofilm formation ability; (**B**) Cell membrane integrity; (**C**) Reactive oxygen species (ROS) levels. All data are presented as mean ± standard deviation (SD) (*n* = 3). Asterisks denote significant differences for each ART treatment versus the control group: * *p* < 0.05, ** *p* < 0.01, *** *p* < 0.001, **** *p* < 0.0001; the absence of markers indicates no significant.

To investigate the mechanism of action of ART against *A. hydrophila* LP-2, its effects on biofilm formation, cell membrane integrity, and intracellular reactive oxygen species (ROS) levels were examined. Quantitative biofilm assays showed that ART significantly inhibited biofilm formation at 400 μg/mL and above, with the inhibitory effect increasing progressively with rising concentrations, whereas no significant biofilm-specific effect was observed at 200 μg/mL (Figure 2A). The cell membrane permeability assay showed that the intracellular fluorescence intensity in the 200 μg/mL ART treatment group was not significantly different from that of the blank control. When the concentration was increased to 400 μg/mL and 800 μg/mL, the fluorescence intensity significantly increased in a concentration-dependent manner, indicating that only moderate and high concentrations of artemisinin increased the permeability of the bacterial cell membrane (Figure 2B). ROS levels followed a trend similar to that of cell membrane integrity, with treatment at 200 μg/mL ART not inducing obvious ROS accumulation, whereas moderate and high concentrations significantly elevated intracellular ROS levels (Figure 2C). These results indicate that ART inhibited *A. hydrophila* LP-2 through distinct mechanisms depending on the concentration range, with growth inhibition at low concentrations and suppression of biofilm formation, disruption of membrane barrier function, and induction of ROS accumulation at high concentrations.

### 3.3. Global Proteomic Changes in Strain LP-2 Induced by Artemisinin

**Figure 3:**
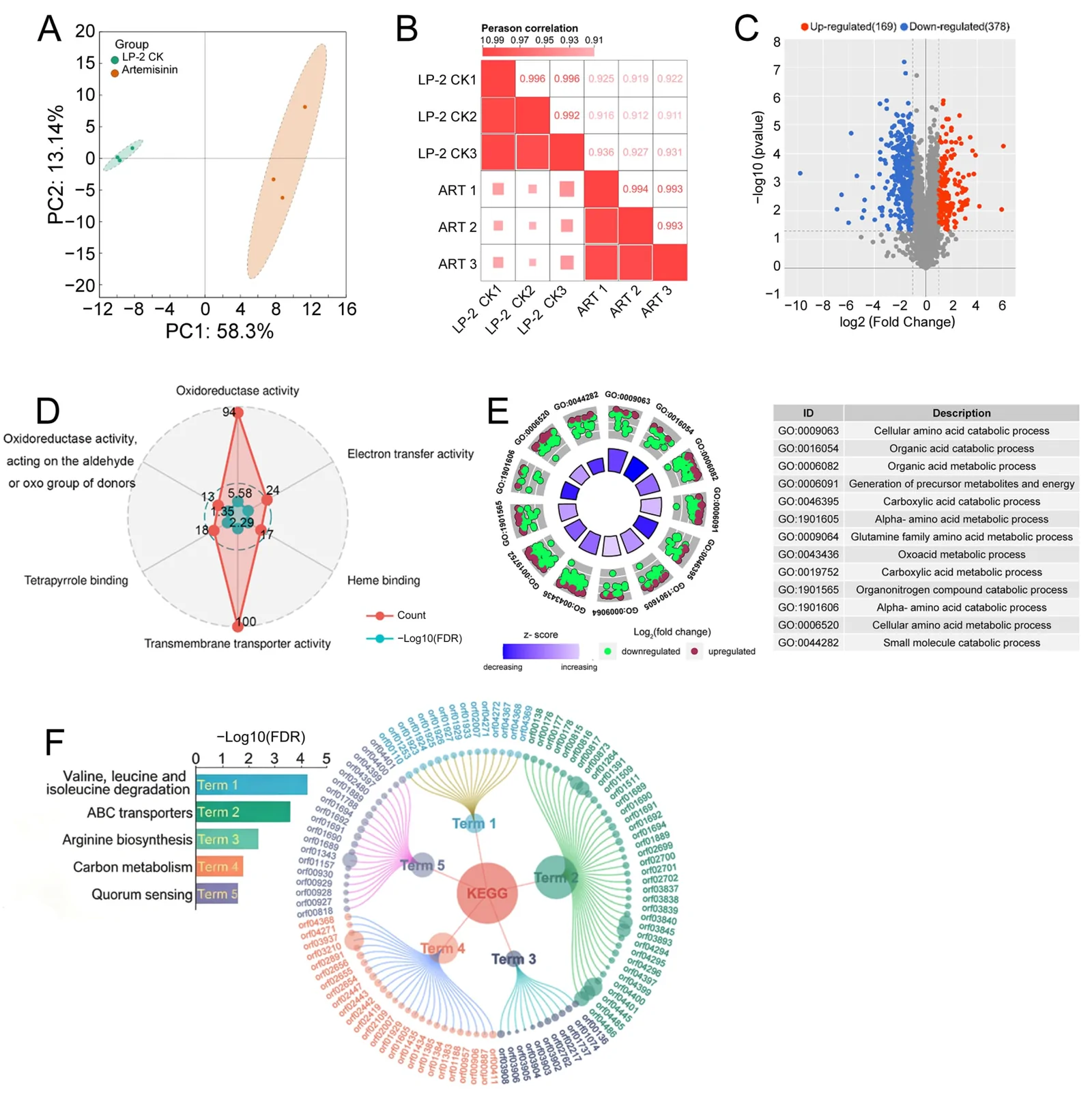
Global proteomic analysis of differentially expressed proteins in *A. hydrophila* LP-2 treated with artemisinin. (**A**) Sample correlation heatmap; (**B**) PCA clustering plot; (**C**) Volcano plot of differentially expressed proteins, with red, blue, and gray dots representing significantly upregulated, downregulated, and non-significant proteins, respectively; (**D**) Radar plot of GO molecular function enrichment, with red and green dots indicating protein count and −log_10_(FDR), respectively; (**E**) Ring plot of GO biological process enrichment, where the inner ring shows the enrichment Z-score and the outer ring displays upregulated (red) and downregulated (green) proteins arranged by log_2_(fold change); (**F**) Chord diagram of KEGG pathway enrichment, with central nodes representing enriched pathways and peripheral nodes representing associated proteins, where node size reflects fold change and the bar chart indicates pathway significance.

To further elucidate the molecular mechanisms by which ART inhibits *A. hydrophila* LP-2, label-free quantitative proteomic analysis was performed on both the untreated control group (CK) and the group treated with 200 μg/mL ART. The sample correlation heatmap showed that the Pearson correlation coefficients among biological replicates within each group were greater than 0.99, which were notably higher than those between groups (0.91–0.93), indicating good reproducibility of the proteomic data and suitability for subsequent differential analysis (Figure 3A). PCA further showed that the CK and ART-treated groups formed two clearly separated clusters, indicating that ART treatment markedly changed the global protein expression profile of LP-2 (Figure 3B). Volcano plot analysis identified a total of 547 differentially expressed proteins under the criteria of |log_2_FC| > 1 and *p* < 0.05, of which 378 were downregulated and 169 were upregulated (Figure 3C). The full quantitative information for all detected proteins is provided in Table S2.

GO enrichment analysis revealed that ART significantly enriched molecular functions including oxidoreductase activity, transmembrane transporter activity, electron transfer activity, heme binding, and tetrapyrrole binding. At the biological process level, artemisinin predominantly downregulated processes related to organic acid catabolism, carboxylic acid catabolism, and alpha-amino acid catabolism, while mainly upregulating processes involved in generation of precursor metabolites and energy, glutamine family amino acid metabolism, organonitrogen compound catabolism, and cellular amino acid metabolism. Consistently, KEGG enrichment analysis identified five pathways, namely valine, leucine and isoleucine degradation, ABC transporters, arginine biosynthesis, carbon metabolism, and quorum sensing (Figure 3D–F). Collectively, these proteomic alterations indicate that artemisinin disrupted bacterial redox metabolism, amino acid metabolism, and transmembrane transport, which may correlate with the observed reductions in bacterial growth and biofilm formation.

**Figure 4:**
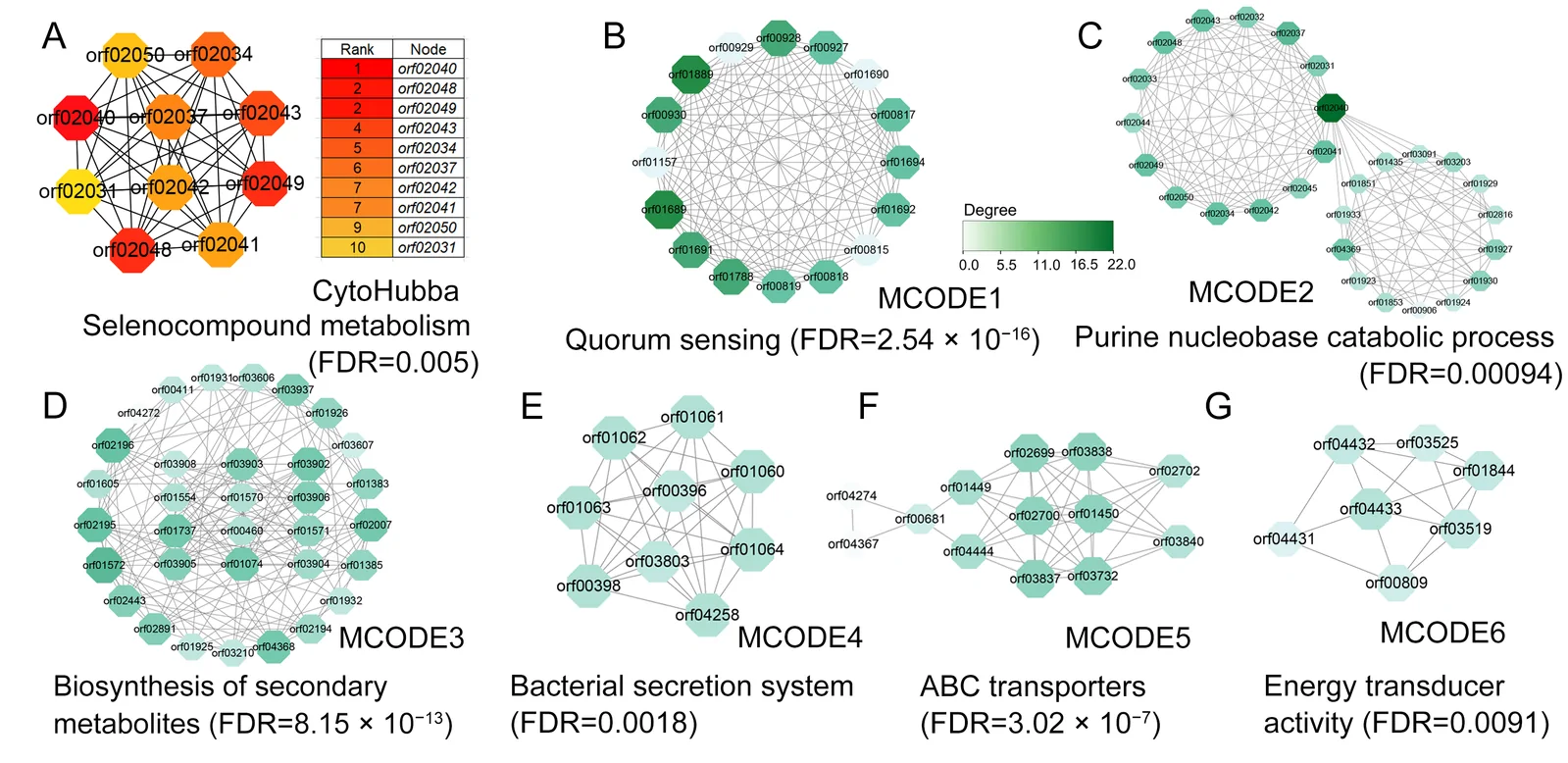
Key nodes and functional modules in the protein–protein interaction (PPI) network. (**A**) Top 10 hub nodes identified by the Maximal Clique Centrality (MCC) algorithm of the CytoHubba plugin. Node color indicates the MCC score, with red representing higher values. (**B**–**G**) Top 6 representative modules identified by the MCODE algorithm and their functional enrichment analysis. The color of protein nodes reflects the degree (connectivity), with darker colors indicating higher degrees.

Furthermore, the protein–protein interaction (PPI) network of differentially expressed proteins was predicted using the STRING database and constructed and analyzed using Cytoscape software(version 3.10.2). Based on the Maximal Clique Centrality (MCC) algorithm of the CytoHubba plugin, 10 key hub proteins were identified, including xanthine dehydrogenase (*orf02043*) and oxidoreductase (*orf02040*) (Figure 4A). These key proteins were all down-regulated and significantly enriched in pathways such as selenium compound metabolism and bacterial secretion system, indicating that they collectively constitute a regulatory network for the strain’s response to ART stress. ART may interfere with related metabolism by inhibiting the expression of these key proteins, thereby inhibiting the growth of the strain. Further modular clustering analysis of the interaction network based on the MCODE algorithm showed that the top 6 highest-scoring modules were significantly enriched in quorum sensing, purine nucleobase catabolic process, biosynthesis of secondary metabolites, bacterial secretion system, ABC transporters, and energy transducer activity (Figure 4B–G). These results suggest that artemisinin may achieve effective inhibition of *A. hydrophila* by synergistically suppressing the aforementioned key pathways of signal transduction, substance transport, and metabolic biosynthesis through multi-target effects.

### 3.4. Validation of Key Differentially Expressed Proteins

**Figure 5:**
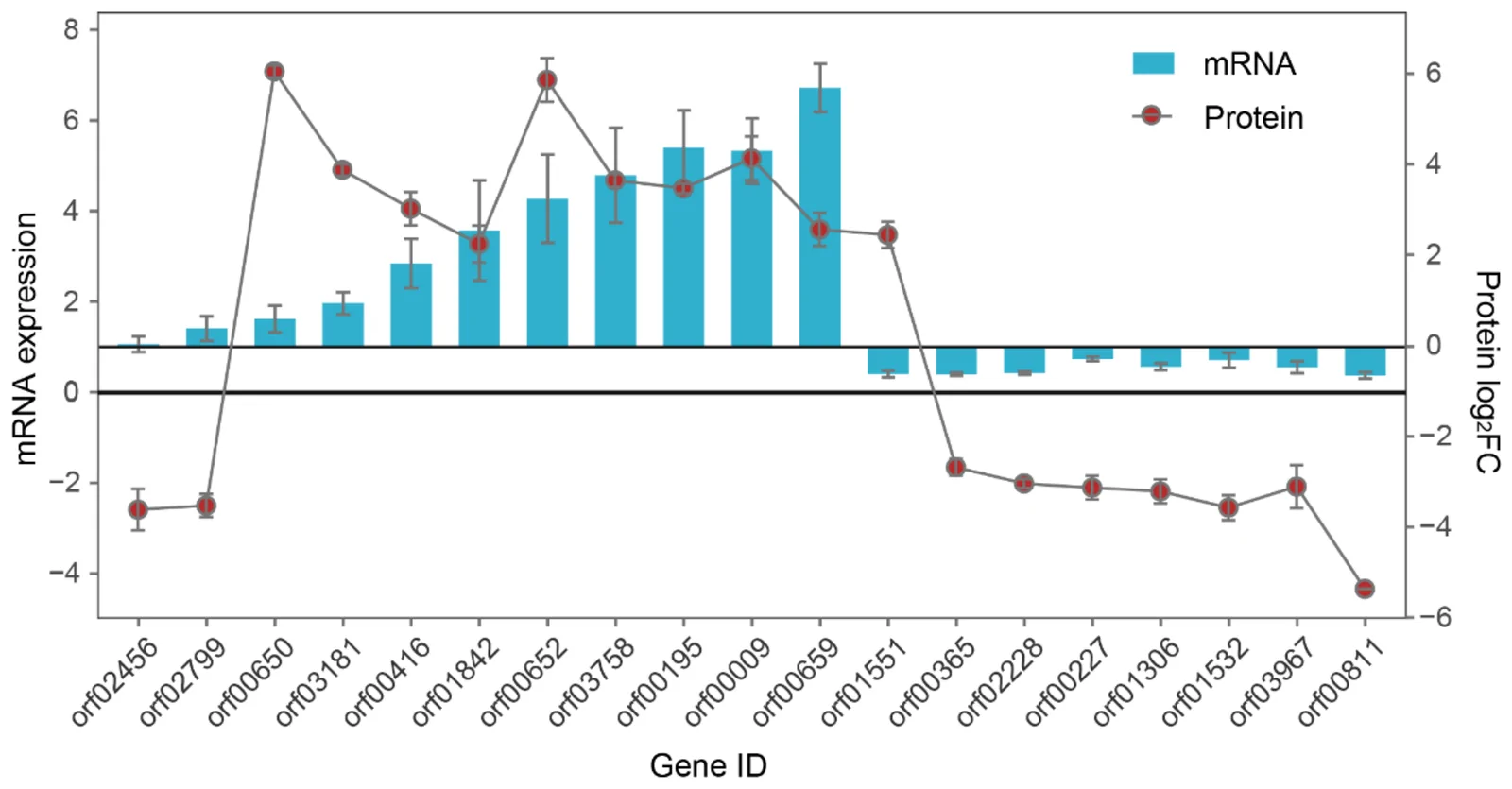
RT-qPCR validation of expression changes of 19 selected genes following ART treatment. Bar plots represent mRNA relative expression from RT-qPCR, and the line plot indicates protein log_2_FC from proteomics.

Given the significant enrichment of differentially expressed proteins in redox metabolism and transmembrane transport pathways, candidate genes associated with these pathways were selected and validated by RT-qPCR. The results showed that ART treatment significantly altered the transcript levels of most target genes in *A. hydrophila* (Figure 5). Genes related to oxidative stress and ROS metabolism, including *orf00650*, *orf00652*, *orf03758*, and *orf00195*, were significantly upregulated, while genes associated with the respiratory chain and electron transfer, including *orf00365*, *orf02228*, *orf00227*, *orf01306*, and *orf01532*, were significantly downregulated. Regarding metal ion homeostasis and transport, genes such as *orf03181*, *orf00416*, and *orf01842*, were significantly upregulated, whereas *orf03967* and *orf00811* were significantly downregulated. Additionally, heat shock protein *orf00009* and multidrug efflux transporter *orf00659* were also significantly upregulated. The transcriptional trends of these genes were consistent with the proteomic data. However, the transcript levels of *orf02456*, *orf01551*, and *orf02799* showed inconsistent trends compared with the proteomic results. Overall, the transcriptional changes observed by RT-qPCR were largely in agreement with the proteomic data, confirming the reliability of the proteomic findings.

### 3.5. Antibacterial Activity of Artemisinin in Combination with Other Compounds

**Figure 6:**
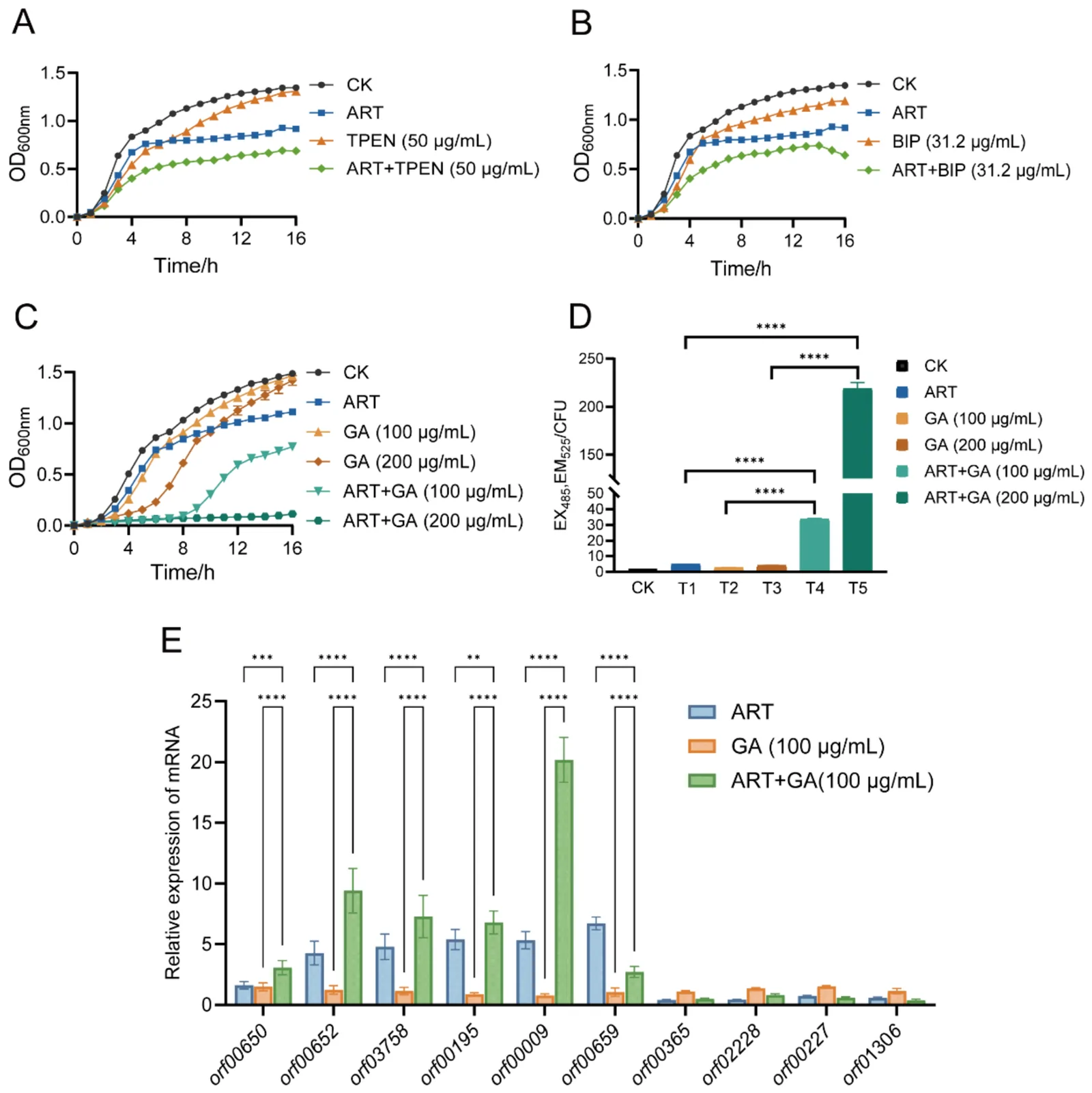
Effects of artemisinin combined with different compounds on the growth and intracellular reactive oxygen species (ROS) levels of *A. hydrophila*. (**A**) Growth curves of LP-2 treated with ART combined with TPEN. (**B**) Growth curves of LP-2 treated with ART combined with BIP. (**C**) Growth curves of LP-2 treated with ART combined with GA. (**D**) Intracellular ROS levels of LP-2 treated with ART combined with GA. Treatments included CK (untreated LP-2), T1 (ART, 200 μg/mL), T2 (GA, 100 μg/mL), T3 (GA, 200 μg/mL), T4 (ART 200 μg/mL + GA 100 μg/mL), and T5 (ART 200 μg/mL + GA 200 μg/mL). For better visualization of low-value groups, the *Y*-axis is broken in panel (**D**). (**E**) Relative mRNA expression levels of related genes in *A. hydrophila* under ART (200 μg/mL), GA or their combination treatments. Data in all panels are presented as mean ± SD (*n* = 3). Asterisks denote significant differences between groups (* *p* < 0.05, ** *p* < 0.01, *** *p* < 0.001, **** *p* < 0.0001), while the absence of markers indicates no significant difference.

To investigate the combined antibacterial effects of ART with TPEN, BIP, or GA, the growth curves of *A. hydrophila* LP-2 under individual and combination treatments were measured. Individual treatment assays showed that TPEN and BIP only mildly inhibited LP-2 proliferation, meanwhile GA also exhibited limited antibacterial activity, merely delaying the growth rate at 200 μg/mL. Combination treatments revealed that all three compounds enhanced the antibacterial activity of ART, but with markedly different synergistic potentials. TPEN and BIP showed weak combined inhibitory effects; 100 μg/mL GA combined with ART significantly delayed bacterial growth and reduced the maximum biomass; and 200 μg/mL GA combined with ART almost completely blocked bacterial growth, achieving the optimal combined effect (Figure 6A–C).

Based on the above phenotypes, we further measured the intracellular reactive oxygen species (ROS) levels in LP-2 following individual or combined treatments with GA and ART. The results showed that intracellular ROS levels after individual treatments were comparable to the control group, whereas the combined treatment induced substantial ROS accumulation, suggesting that the combined antibacterial effect may be mediated by oxidative stress (Figure 6D).

To further elucidate the molecular mechanism underlying this synergy, RT-qPCR was performed to analyze the transcriptional changes in genes related to ROS metabolism and oxidative stress in LP-2 under ART, GA, or their combination. The results indicated that GA alone hardly altered the expression of the target genes (Figure 6E). Compared with ART alone, the combined treatment significantly upregulated the mRNA expression of *orf00650* (Putative oxidoreductase YncB), *orf00652* (Organic hydroperoxide resistance protein), *orf03758* (NADH-dependent flavin oxidoreductase, Oye family), *orf00195* (Hypothetical oxidoreductase YqhD), and *orf00009* (16 kDa heat shock protein A), while significantly downregulating the expression of *orf00659* (Multidrug efflux transporter, MFS family). The upregulated genes mainly encode oxidoreductases and heat shock proteins, indicating aggravated oxidative stress; conversely, the downregulated gene encodes an MFS-family efflux transporter, suggesting a potential impairment of the bacterial drug efflux defense. The combined effect of intensified oxidative stress and weakened efflux defense may represent an important mechanism for the observed antibacterial effect. Furthermore, considering that the respiratory chain is a major source of intracellular ROS, the transcriptional levels of respiratory chain-related genes (e.g., *orf00365* (ubiquinol cytochrome C oxidoreductase), *orf02228* (Cytochrome c oxidase subunit CcoP), *orf00227* (Cytochrome c-type protein TorY), and *orf01306* (Putative diheme cytochrome c-553)) were also examined. The results showed no significant differences between the combined and individual treatments, suggesting that this combined effect may not be primarily achieved through transcriptional regulation of respiratory chain-related genes.

### 3.6. Hemolytic Activity of Artemisinin

**Figure 7:**
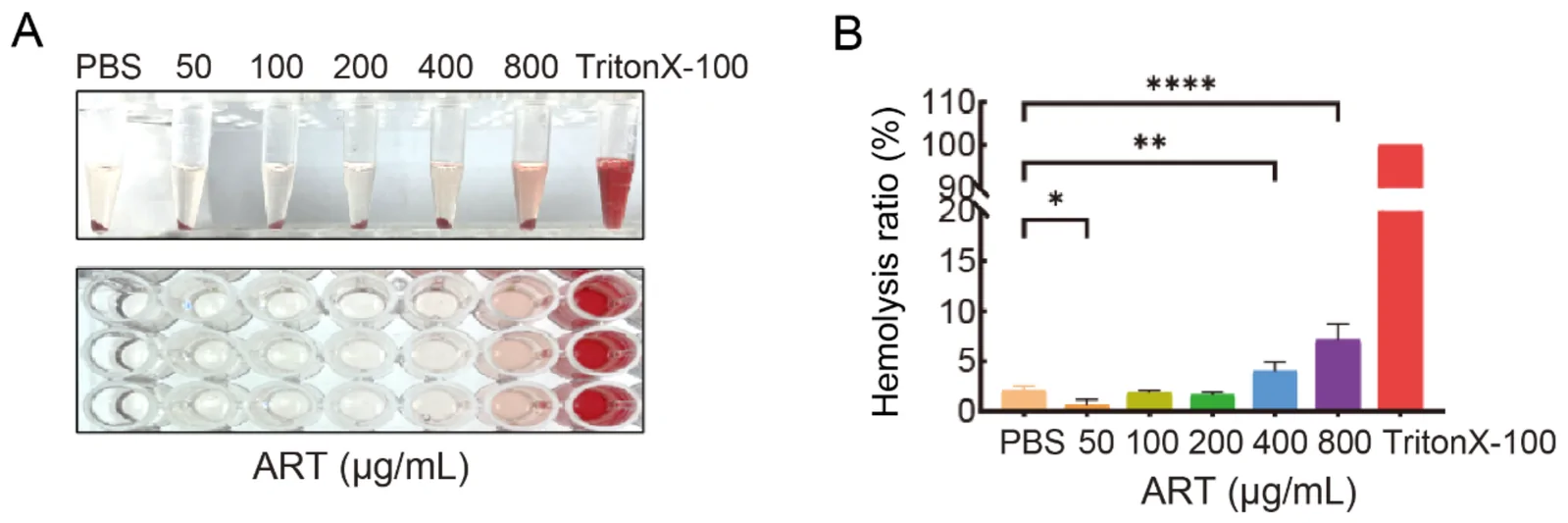
In vitro hemolytic activity of artemisinin against red blood cells. (**A**) Qualitative observation of the hemolysis assay; (**B**) Comparison of hemolysis rates under different concentrations of ART. Triton X-100 served as the positive control for 100% hemolysis. All data are presented as mean ± standard deviation (SD) (*n* = 3). Data were analyzed by one-way ANOVA with Tukey’s test. Asterisks denote significant differences between groups (* *p* < 0.05, ** *p* < 0.01, *** *p* < 0.001, **** *p* < 0.0001), while the absence of markers indicates no significant difference.

Given the notable antibacterial activity of ART against *A. hydrophila*, its potential hemolytic risk as a candidate agent was further evaluated. The results showed that ART did not induce obvious hemolysis of red blood cells in vitro within the concentration range of 50 to 200 μg/mL. Although slight hemolysis was observed at high concentrations of 400 and 800 μg/mL, the hemolysis rates in both treatments were below 10%, which were significantly different from that of the Triton X-100 positive control (Figure 7). These results indicate that ART exhibits no hemolytic toxicity. However, its relevance to aquaculture species requires further validation using fish erythrocytes or in vivo models.

## 4. Discussion

Artemisinin, a sesquiterpene lactone compound containing an endoperoxide bridge, displays marked differences in its antibacterial activity against different bacterial species. For instance, it shows strong inhibition against Gram-positive bacteria such as *Staphylococcus aureus* and *Bacillus subtilis*, but limited efficacy against Gram-negative bacteria like *E. coli* and *P. aeruginosa* [14,25]. The present study further confirmed that artemisinin exhibited pronounced inhibitory activity against *A. hydrophila*, whereas its effects on the tested *Vibrio* and *Pseudomonas* species were relatively weak. This species-specific antibacterial activity may be associated with the composition and regulatory status of bacterial RND-type efflux systems. It has been reported that inactivation of AtrR in *Vibrio cholerae* upregulates the VexAB–TolC system, leading to active efflux of artemisinin and subsequent resistance; similarly, *P. aeruginosa* harbors broad-spectrum RND efflux pumps such as MexAB–OprM, which may restrict intracellular accumulation of artemisinin [26]. Although these findings are largely derived from model strains or mutants, the weak inhibitory phenotype observed in the tested *Vibrio* and *Pseudomonas* species in this study may still be attributable to the presence of specific RND efflux pumps or elevated expression levels of related efflux genes.

Based on the species-specific observations above, our study focused on the most sensitive strain, *A. hydrophila* LP-2, to investigate the antibacterial mechanism of artemisinin. Phenotypic assays showed that low concentration (200 μg/mL) of artemisinin induced limited phenotypic changes, whereas high concentrations (400 μg/mL and 800 μg/mL) triggered biofilm disruption, intracellular ROS accumulation and increased membrane permeability, which may affect membrane barrier function. These changes are largely consistent with previous reports, as artemisinin-type compounds can interfere with biofilm formation at sub-inhibitory concentrations [27,28], and artemisinin induces peroxides in a time- and concentration-dependent manner accompanied by rapid membrane disintegration [29]. It should be noted that ethanol alone also showed an effect at the level accompanying 800 μg/mL artemisinin, and this solvent contribution needs to be considered. Collectively, these findings indicate that artemisinin exerts a multi-stage antibacterial effect, with its specific mechanism varying across concentrations.

To further investigate the antibacterial mechanism of artemisinin against *A. hydrophila*, quantitative proteomics was applied, indicating that artemisinin treatment was associated with changes in multiple biological pathways, including redox processes, purine catabolism, ABC transporter systems, and quorum sensing. Within these pathways, key proteins such as XDH family oxidoreductases were significantly downregulated. These molecular alterations were consistent with the observed phenotypes of reduced growth rate and impaired biofilm formation. Notably, the ABC transporter system and quorum sensing (QS) pathway have been frequently reported as key targets of natural products, as demonstrated by paeonol, which downregulates ABC transporter proteins in *A. hydrophila* [30]. Mechanistically, the ABC transporter system mediates the uptake of amino acids and oligopeptides; its inhibition restricts nutrient supply and protein synthesis, thereby suppressing bacterial growth. Similarly, the AhyI-AhyR quorum sensing pathway positively regulates biofilm matrix synthesis, and its disruption impairs biofilm formation [31,32]. In contrast, natural products that target purine catabolism remain poorly characterized [33,34,35]. Given the role of purine metabolism in biofilm development, the function of XDH family proteins in artemisinin-mediated inhibition of *A. hydrophila* warrants further investigation [36,37].

Artemisinin (and its derivatives) in combination with other active molecules exhibit significant antibacterial effects. Previous studies have shown that dihydroartemisinin–chitosan nanoparticles disrupt bacterial membranes and promote intracellular drug accumulation. Once inside the cell, intracellular Fe^2+^ and Cu^+^ catalyze endoperoxide bridge cleavage, inducing massive ROS accumulation and DNA damage, thereby enhancing antibacterial activity [15,16]. Our study showed that the artemisinin–gallic acid (ART + GA) combination exhibited significantly superior antibacterial activity compared with artemisinin combined with TPEN or BIP. This difference may be attributed to the distinct metal-binding properties of these molecules. TPEN and BIP are high-affinity ligands that sequester intracellular free Fe^2+^/Cu^+^, thereby potentially blocking the metal-dependent activation of artemisinin. In contrast, gallic acid, as a moderate-affinity ligand, can bind metal ions without completely eliminating their redox activity [38,39,40]. This suggests that distinct metal-coordination modes may account for the variation in antibacterial activity among these combinations.

In investigating the possible mechanism underlying the synergistic effect at the level of growth inhibition of the ART + GA combination, further analysis showed that the intracellular ROS level in the combination group was significantly higher than that in each individual treatment. Additionally, oxidative stress defense-related genes (*orf00650*, *orf00652* and *orf00009*) were upregulated, while the multidrug efflux gene (*orf00659*) was downregulated. These observations are consistent with the antibacterial mechanism reported for GA combined with caffeic acid, which also involves ROS accumulation accompanied by upregulation of antioxidant enzymes and efflux systems at the protein level [41]. Thus, efflux inhibition and oxidative stress response may represent a common mechanism underlying the antibacterial activity of GA-involved combinations.

With respect to efflux inhibition, previous studies have shown that gallic acid alone exhibits limited antibacterial activity, yet it can enhance antibiotic efficacy by inhibiting bacterial efflux pumps when used in combination. For instance, GA combined with tetracycline inhibits the MFS efflux pump in *Staphylococcus aureus*, and its combination with isoniazid and rifampicin significantly suppresses the expression of MFS efflux pump genes in *Mycobacterium tuberculosis* [42,43]. Given that *orf00659* is annotated as an MFS family multidrug efflux transporter, it is conceivable that GA downregulates the expression of this MFS efflux transporter, thereby potentially increasing the effective accumulation of ART within bacterial cells. It should be noted, however, that whether *orf00659* directly effluxes ART requires further experimental validation.

With respect to oxidative stress, GA in combination with antibiotics such as azithromycin has been shown to significantly elevate intracellular ROS levels in bacteria [44]. This effect has been proposed to be associated with the ability of gallic acid to continuously convert Fe^3+^ to Fe^2+^ via single electron transfer, thereby providing a sustained supply of Fe^2+^ for both Fenton-like reactions and artemisinin activation [45]. It is therefore reasonable to propose that, in the ART + GA combination, the enhanced elevation of ROS is consistent with the combined generation of ROS via Fenton reactions and artemisinin-derived free radical production, while the upregulation of *orf00650*, *orf00652*, and *orf00009* may represent a compensatory stress response elicited by *A. hydrophila* to maintain intracellular redox homeostasis. These findings support a model in which GA, through its redox-cycling of Fe^2+^, potentiates artemisinin-mediated oxidative stress; nevertheless, further studies, including ROS-scavenger rescue experiments, cell-free probe controls, direct measurement of intracellular iron levels, and Fe^2+^/Fe^3+^ supplementation experiments, are warranted to establish the direct contribution of the GA–Fe–Fenton cycle to the enhanced antibacterial activity.

## 5. Conclusions

In summary, this study demonstrates that artemisinin exerts multi-target antibacterial effects against *A. hydrophila* LP-2, including biofilm inhibition and ROS accumulation. The artemisinin-gallic acid (ART + GA) combination exhibits significant inhibitory activity, which is associated with upregulation of oxidative stress defense genes and downregulation of the MFS efflux gene *orf00659*, without apparent hemolytic toxicity at the tested concentrations. Based on these phenotypic and transcriptional observations, we propose a putative mechanism in which GA-mediated efflux suppression and moderate metal-coordination properties may synergistically enhance ART’s antibacterial action. However, it must be emphasized that direct evidence for ART binding to its putative targets, the causal link between efflux inhibition and ROS elevation, the precise role of metal chelation, and the direct effects on ABC transporter function and quorum-sensing activity remain lacking and requires future experimental validation.

## Data Availability

The mass spectrometry proteomics data have been deposited in the ProteomeXchange Consortium via the iProX partner repository with the dataset identifier PXD084852.

## Supplementary Materials

The following supporting information can be downloaded at: https://www.mdpi.com/article/10.3390/biology15212008/s1, Table S1. Primers used for RT-qPCR validation of selected differentially expressed genes. Table S2. Summary of all quantified proteins in *A. hydrophila* LP-2 with or without artemisinin treatment.

## Abbreviations

The following abbreviations are used in this manuscript:

ART	artemisinin
GA	gallic acid
TPEN	N,N,N′,N′-tetrakis(2-pyridylmethyl)ethylenediamine
BIP	2,2′-bipyridine
ROS	reactive oxygen species
MFS	major facilitator superfamily
